# Quantitative evaluation of lower limb varicose veins using photoacoustic imaging

**DOI:** 10.1007/s10396-024-01470-8

**Published:** 2024-06-20

**Authors:** Moemi Urano, Kenichi Nagae, Sachiko Matsuda, Kentaro Matsubara, Takayuki Yagi, Nobuaki Imanishi, Sadakazu Aiso, Hideaki Obara, Masahiro Jinzaki

**Affiliations:** 1 Department of Radiology, Keio University School of Medicine, Tokyo, Japan; 2 Department of Surgery, Keio University School of Medicine, 35 Shinanomachi, Shinjuku-ku, Tokyo, 160-8582 Japan; 3 Department of Anatomy, Keio University School of Medicine, Tokyo, Japan; 4 Luxonus Inc., Kawasaki, Kanagawa Japan

**Keywords:** Photoacoustic imaging, Varicose vein, Blood vessel, Heat map

## Abstract

**Purpose:**

Varicose veins in the lower extremities are dilated subcutaneous varicose veins with a diameter of  ≥ 3 mm, caused by increased venous pressure resulting from backflow of blood due to venous valve insufficiency (Gloviczki in Handbook of venous disorders: guidelines of the American venous forum, Hodder Arnold, London, 2009). When diagnosing varicose veins, the shape and thickness of the blood vessels should be accurately visualized in three dimensions. In this study, we investigated a new method for numerical evaluation of vascular morphology related to varicose veins in the lower extremities, using a photoacoustic imaging (PAI) system, which can acquire high-resolution and three-dimensional images noninvasively.

**Methods:**

Nine patients with varicose veins participated in the study, and their images were captured using an optical camera and PAI system. We visualized the vascular structure, created a blood presence density (BPD) heat map, and examined the correlation between BPD and location of varicose veins.

**Results:**

The obtained photoacoustic (PA) images demonstrated the ability of this method to visualize vessels ranging from as small as 0.2 mm in diameter to large, dilated vessels in three dimensions. Furthermore, the study revealed a correlation between the high-density part of the BPD heat map generated from the PAI images and the presence of varicose veins.

**Conclusion:**

PAI is a promising technique for noninvasive and accurate diagnosis of varicose veins in the lower extremities. By providing valuable information on the morphology and hemodynamics of the varicose veins, PAI may facilitate their early detection and treatment.

## Introduction

Varicose veins are a common disease with a prevalence of 20–60% in the global population, and approximately 5% of affected individuals require treatment for their symptoms [2–5]. Varicose veins are caused by the failure of valves in the veins, which results in a lack of venous blood flow in the normal direction, eventually causing reflux and stagnation of blood and leading to vascular dilation, tortuosity, and varicosities.

Varicose veins can worsen to more advanced stages of chronic venous dysfunction such as chronic venous insufficiency (CVI) [6, 7]. Progression of this condition leads to pain, swelling, itching, bleeding, and skin changes, increasing the risk of thrombosis and ulceration [1, 8]. In other words, varicose veins do not directly cause death; however, they significantly reduce the quality of life of affected patients [2, 9, 10], and do not heal spontaneously. The CEAP clinical classification, which documents the clinical class, etiology, anatomy, and pathophysiology (CEAP) of chronic venous disease (CVD), is recommended [11, 12]. The severity of symptoms and prevalence of symptomatic patients increase proportionally with the CEAP clinical class (C0 to C6) of CVD [13]. Diagnosis includes visual inspection of vascular dilation, pigmentation, and ulcers, and assessment of symptoms such as pain, edema, heaviness, and itching by means of history taking. In addition, the reflux and vein diameters are measured and classified using ultrasonography. The CEAP classification system categorizes varicose veins based on their clinical, etiological, anatomical, and pathophysiological features. This classification system is useful in assessing the severity and outcomes of varicose veins. However, studies on intraobserver and interobserver variabilities have shown significant discrepancies in the CEAP clinical classifications [14–16]. Therefore, clearer and more objective criteria are needed.

The photoacoustic technique can visualize the absorbers in the body through the photoacoustic effect [17]. By varying the wavelength, absorbers such as blood vessels and dyes can be selectively visualized. Various systems using this technique have been developed, and clinical studies using these techniques are ongoing [18–22].

In this study, we investigated a novel index, blood presence density (BPD), which is derived from PA images, to quantify the vascular morphology alterations induced by lower extremity varicose veins. We also investigated the correlation between this index and the clinical diagnosis.

## Materials and methods

### Patients

Eleven patients who visited Keio University Hospital between March 2017 and March 2021 were enrolled in this study. After two patients were excluded due to insufficient data, nine patients (three males and six females; a total of 12 feet) were ultimately included in this study. The mean age of the patients was 70.8 ± 11.5 years.

### Imaging methods for PA imaging

In this study, after the diagnosis of varicose veins in the lower extremities (history taking, visual inspection, palpation, and echography), we captured images of the lower extremities using an optical camera and a PAI system. When imaging with the PA imaging device, we placed the lower extremity medially in the prone position or the calf in the supine position within the imaging range and imaged them.

We used the PAI-05 (Luxonus Inc., Kanagawa, Japan [23]), which is equipped with a hemispherical detector array that enables 3D visualization of blood vessels, to observe the course of thin vessels in a wide range in three dimensions. This device can visualize blood vessels in the body with a spatial resolution of 0.2 mm [24, 25]. In this study, a laser with a wavelength of 797 nm was used to capture images with an imaging area of 270 × 180 mm and an acquisition time of 12 min. These images were output in the Digital Imaging and Communications in Medicine (DICOM) data format and analyzed using MATLAB software (MathWorks Inc.).

### Image processing of camera & PA images

**Fig. 1:**
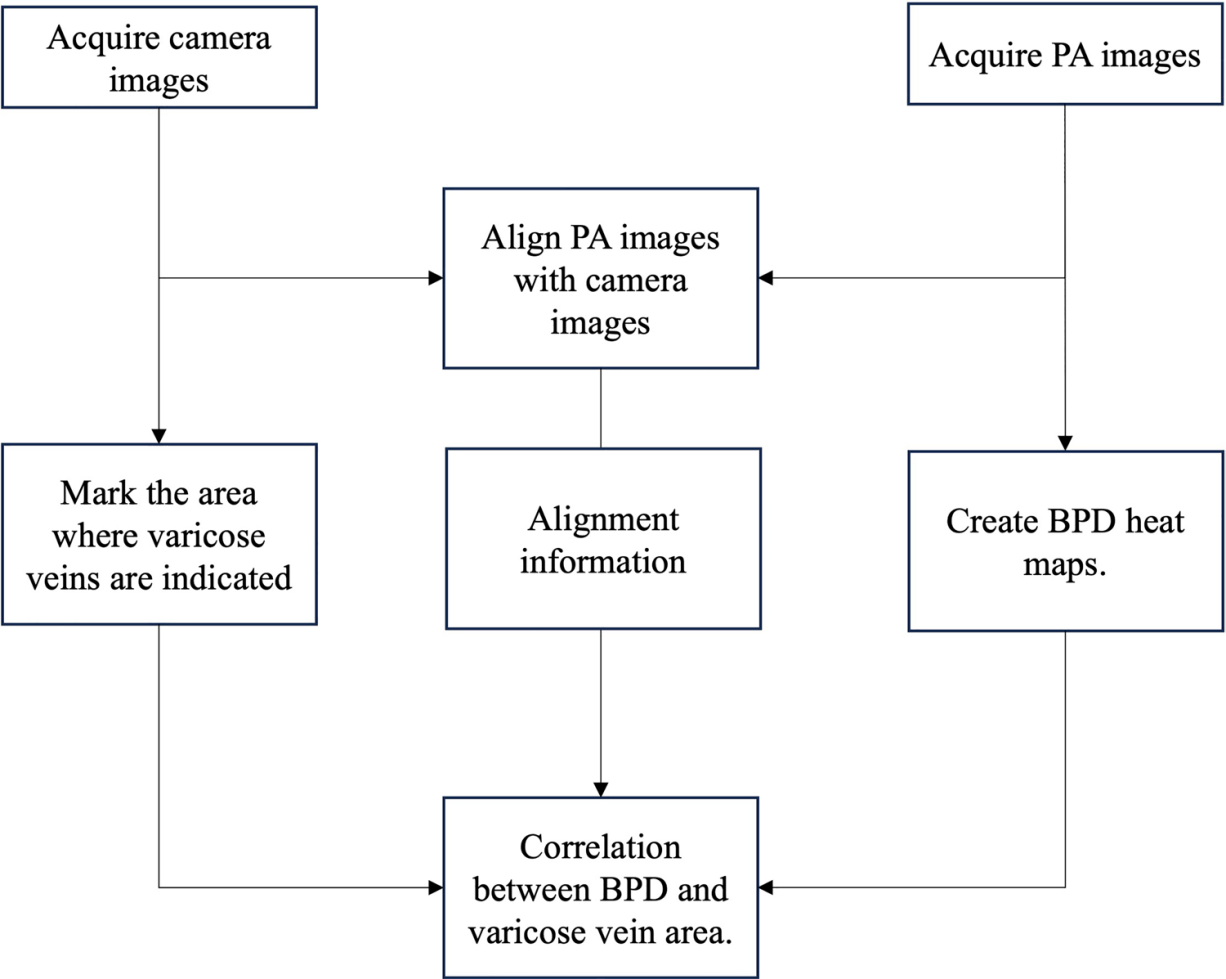
Image processing flow

The acquired camera images and PA images were processed (Fig. 1).

To examine the correlation between the varicose vein marked area and optical ultrasound image, the camera and optical ultrasound images were processed separately, and two images were superimposed for analysis. The details of this process are described below.

#### Processing of camera images

**Fig. 2:**
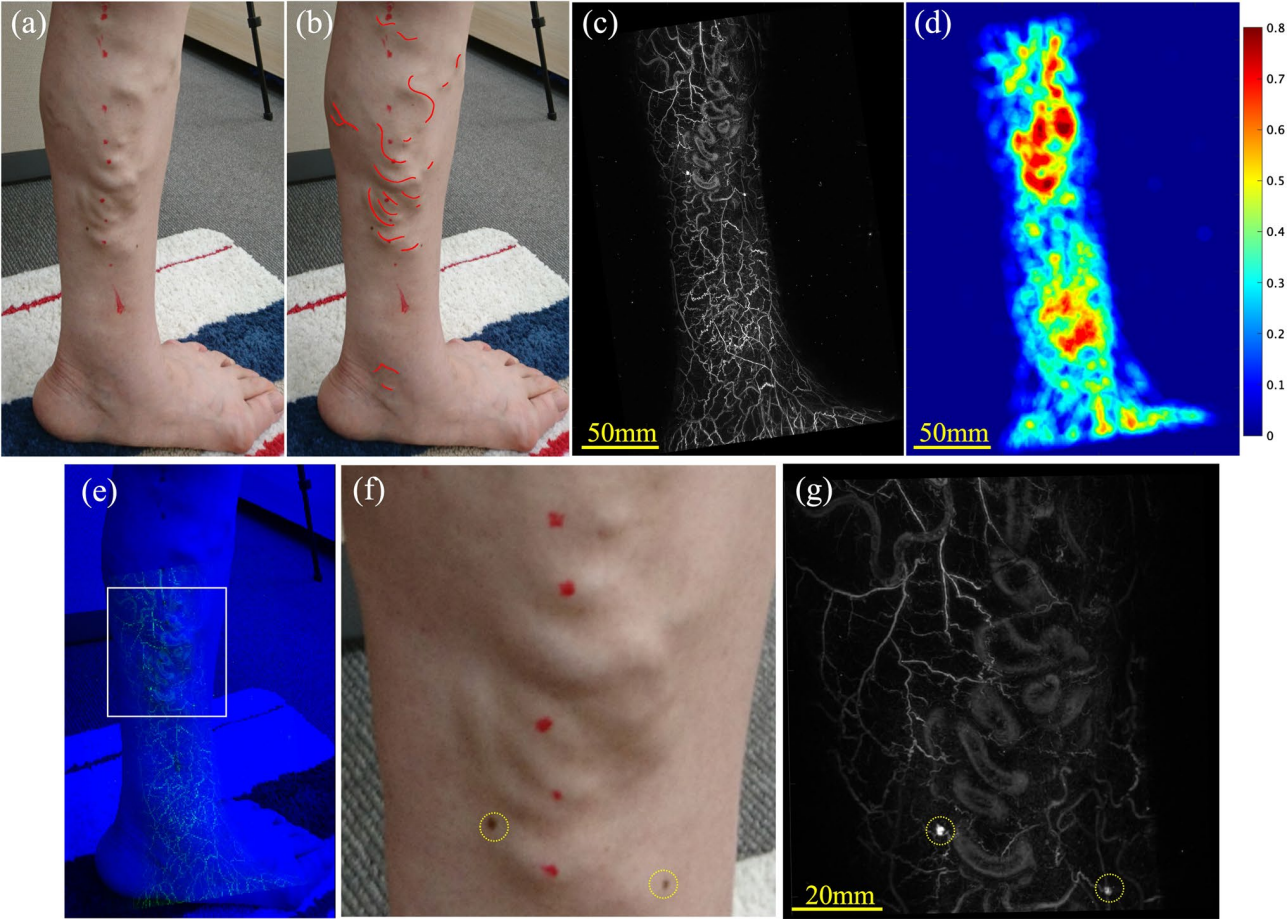
Examples of processing camera images and PA images. **a** Marking of the lower limb varicose vein area by the physician on the camera image. **b** Camera image of the lower limb. **c** PA image of the lower limb. **d** BPD heat map calculated from the PA image. **e** Overlapped image after aligning the camera image and the PA image. **f** Magnified camera image of the white-framed area in (**e**). **g** Magnified PA image of the white-framed area in (**e**). *Example of the morphology of the dilated and tortuous blood vessels in (**f** and **g**), and the mole (yellow dotted line) aligned with the position of the mole (yellow dotted line)

The physician marked the areas (veins) that were visually diagnosed as varicose (Fig. 2a) against the camera image of the lower extremities (Fig. 2b).

#### Processing of PA images

We introduced BPD as an indicator of the possibility of varicose veins in the lower extremities. Varicose veins are caused by impaired venous circulation, which leads to dilation, extension, and formation of varicose veins. Therefore, we assumed that areas of high blood volume per unit volume corresponded to varicose veins. The PA images were processed to calculate the BPD, which represents the distribution of blood volume per unit volume. Maximum Intensity Projection (MIP) images (Fig. 2c) were created by projecting the acquired PA images in two dimensions. Utilizing Otsu’s method [26] across multiple PA images enabled us to identify a threshold for vascular region extraction. This threshold was applied to all PA images to extract the vascular regions. The proportion of the vascular region within a circular area of 10 mm in diameter was visualized as a heat map. The diameter of the circular area was set at 10 mm to facilitate the detection of vascular conditions, including not only dilated vessels but also reticular veins (vessel diameter 2–3 mm) and spider veins (vessel diameter less than 1 mm), as variations in the proportion of the vascular region within the circular area. The mapping results would indicate the distribution of the BPD. The three-dimensional volume of the PA image consisted of voxels with a size of 0.125 mm; therefore, the pixels of the two-dimensional MIP image had a size of 0.125 mm. This distribution is shown in the heat map (Fig. 2d). Here, the number indicated by the color bar (e.g., 0.8) is 80%.

#### Correlation between BPD and varicose vein area

To investigate the correlation between the regions marked as varicose veins and BPD distribution, we performed the following processing on the camera and PA images. First, the camera image before marking and the PA image before heat mapping were aligned to match the characteristic vessel morphology and saphenous vein (Fig. 2e–g), and the alignment information was output. The alignment information of the camera image with markings and the heat map of BPD were obtained using this alignment information. This enabled us to analyze the correlation between the marked positions indicated by the physician as varicose veins and the BPD distribution calculated from the PA images. The BPD in the varicose vein marked area and other areas on the BPD heat map were extracted. The histograms of each area were recorded. Furthermore, as an indicator of correlation, the ratio of the mean BPD of the varicose vein indication area to the total area was calculated.

## Results

### Demographics of study participants

In this study, data from nine patients with varicose veins (12 lower extremities) were analyzed. The mean age of the patients was 70.8 ± 11.5 years; three were male and six were female. The severity of the varicose veins was assessed using the CEAP classification, with two patients having C4, two having C2, and three having C1 veins (Table 1).

**Table 1:** Patient demographics

Patient No	Age [years]	Sex	Lower limb	CEAP
1	72	Male	Right medial	C_2,4,_ E_p,_ A_s,_ P_r, GSVa,GSVb_
2	73	Female	Left medial	C_2,_ E_p,_ A_s_, P_r, GSVa,GSVb_
3	78	Male	Left medial	C_1,2,4a,4b,_ E_p,_ A_s,_ P_r, Ret, GSVa,GSVb_
4	81	Female	Right calf	C_1,_ E_p,_ A_s,_ P_r, Ret_
			Left medial	C_1,4a,_ E_p,_ A_s,_ P_r, Tel, Ret_
5	71	Male	Right medial	C_1,2,_ E_p,_ A_s,_ P_r, Tel, GSVa, GSVb_
6	81	Female	Left calf	C_2,_ E_p,_ A_s,_ P_r, SSV_
7	80	Female	Right medial	C_1,_ E_p,_ A_s,_ P_r, Ret, GSVa, GSVb, SSV_
			Right calf	C_1,_ E_p,_ A_s,_ P_r, Ret, GSVa, GSVb, SSV_
8	53	Female	Left medial	C_1,_ E_p,_ A_s,_ P_r, Ret, GSVa, GSVb, SSV_
			Right calf	C_1,2,_ E_p,_ A_s,_ P_r, Ret, GSVa, GSVb, SSV_
9	48	Female	Right medial	C_1,_ E_p,_ A_s,_ P_r, Tel, Ret_

### Marking the area where varicose veins are indicated

The areas indicated as varicose veins were marked on the camera images by a physician (Fig. 2b).

### PA imaging and BPD heat map

**Fig. 3:**
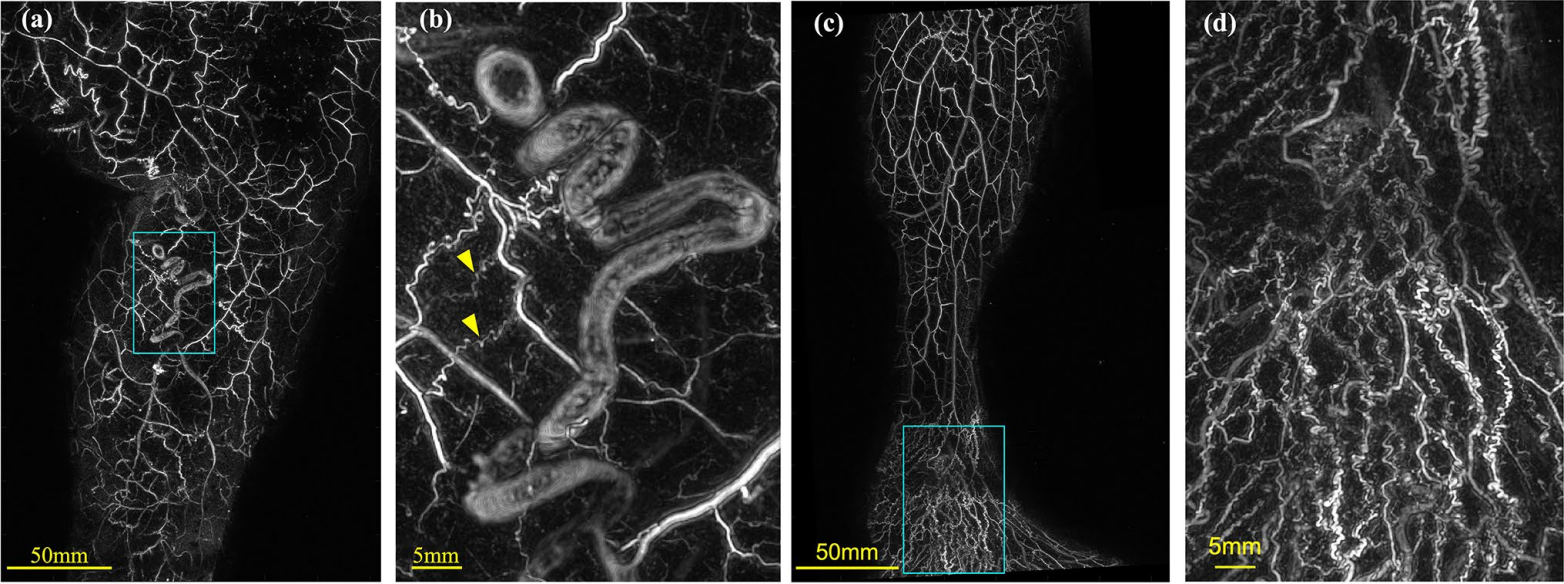
Examples of PA images of the lower limb. **b**, **d** Enlarged image of (**a**, **c**). Various thicknesses of vessels, ranging from thin vessels of about 0.2 mm to dilated vessels, were observed on PA images. The red triangular markers denote the fine vessels, approximately 0.2 mm in diameter

**Fig. 4:**
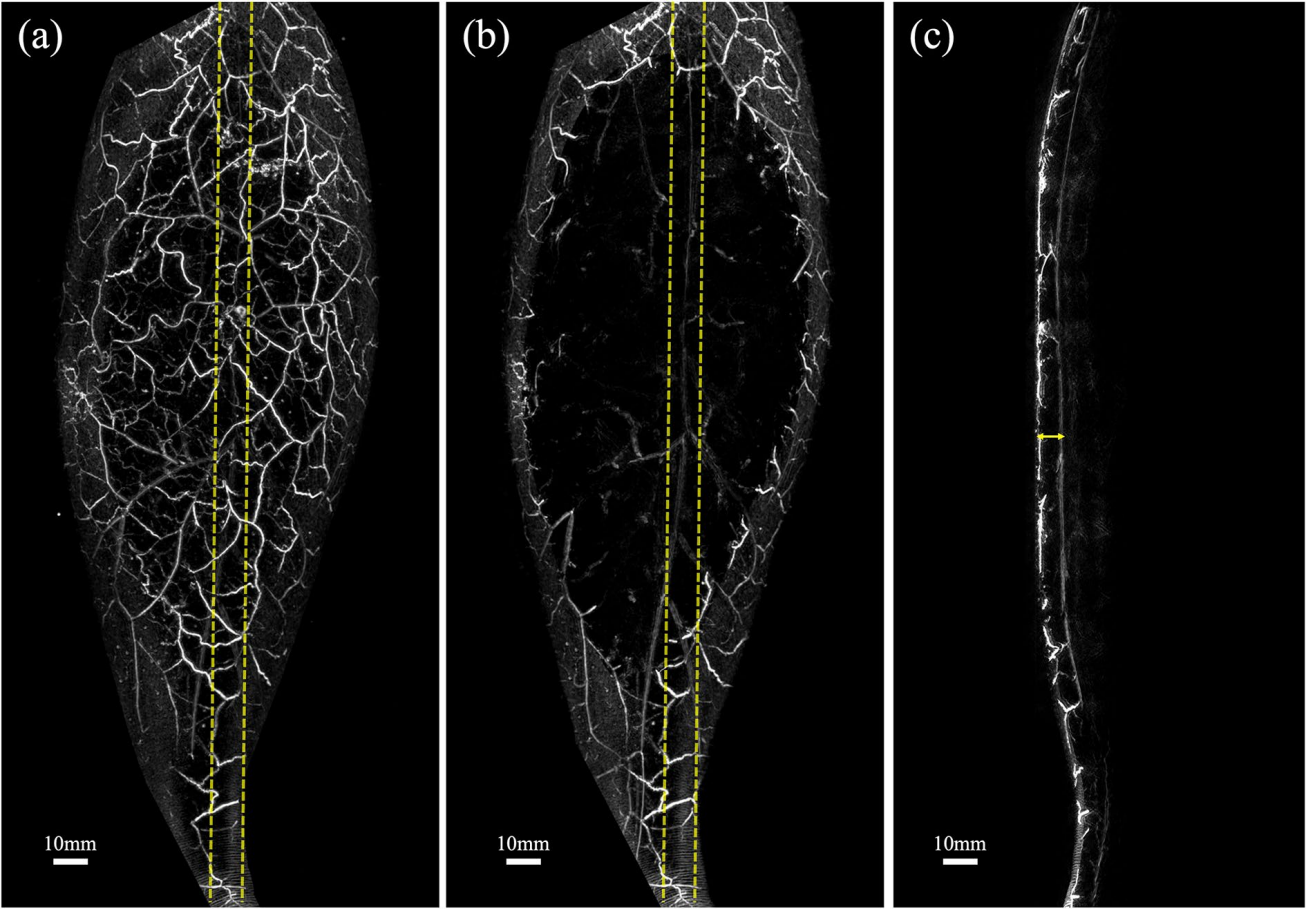
Examples of PA images of the perineum. **b** Display from 6-mm depth from surface (MIP display shaved 6 mm from surface of (**a**). **c** Cross-sectional view of the yellow dotted line in (**a**, **b**). SSV at a depth of about 10 mm from the skin surface can be observed

On the PA images obtained, various vessels, ranging from thin vessels of about 0.2 mm to nodules and dilated vessels, were observed in the lower limb (Fig. 3). These thin finely tortuous vessels were not visible to the naked eye. Moreover, the small saphenous vein (SSV) was observed at a depth of 10 mm (Fig. 4). Thus, from the PA images, we could clearly visualize the three-dimensional vascular structure of the lower limbs affected by varicose veins.

**Fig. 5:**
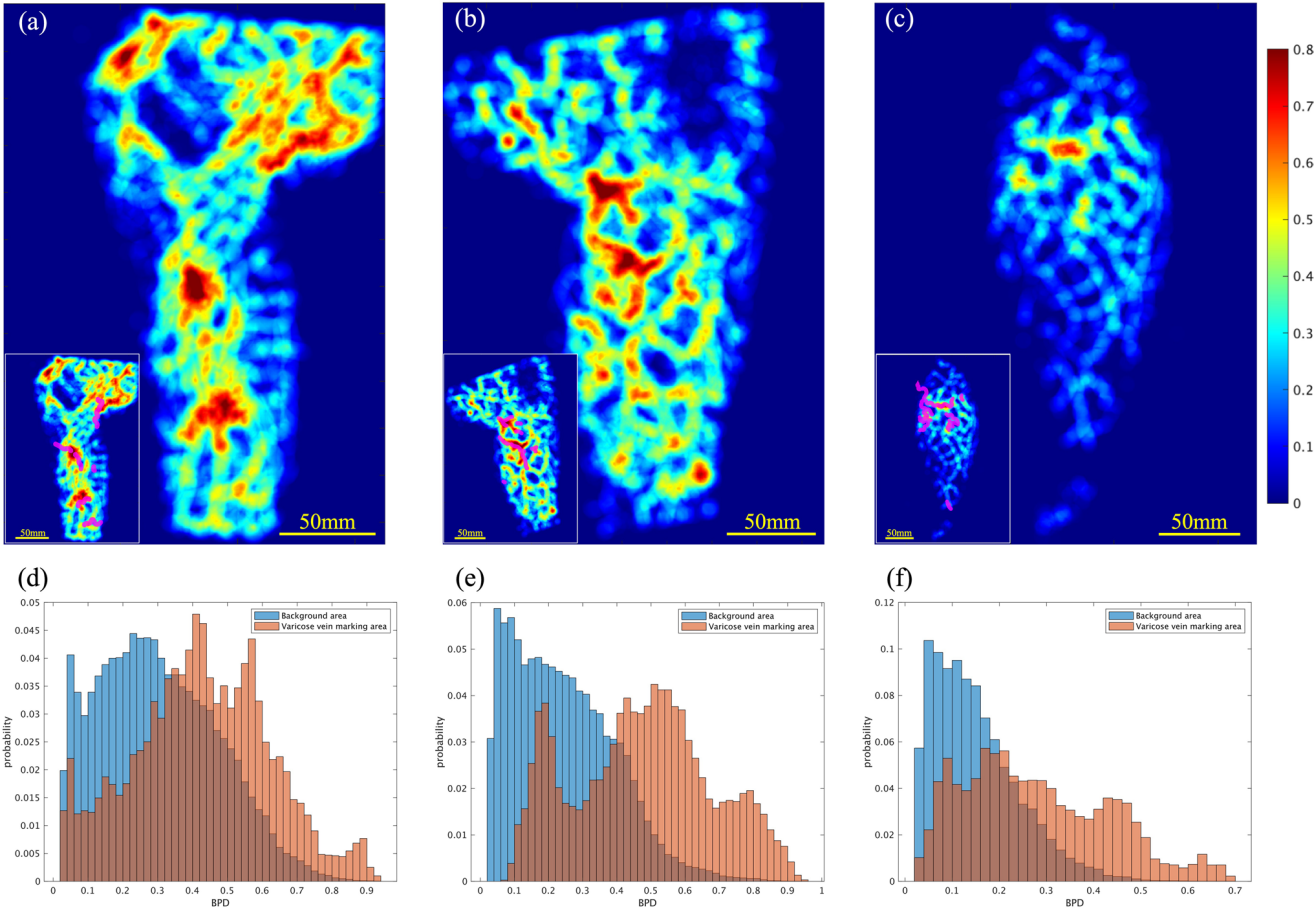
Heat map of BPD generated from PA images (**a**–**c**). The small image in each image is a heat map in which the positions that the physician identfied as varicose veins on the camera image are aligned on the PA image and superimposed with pink lines. **a** Same patient as in Fig. 3a (left medial). **b** Same patient as in Fig. 3c (left medial). **c** Same patient as in Fig. 4 (right perineal). Example of a histogram of BPD in varicose vein marked area and background area, respectively; since the number of elements in the varicose vein marked area and background area are very different, the total area was scaled to match and calculated as a relative percentage. (The number of elements in a bin is divided by the number of elements in the input data) (**d**–**f**). **d** Same patient as in Figs. 3a and 5a (left medial). **e** Same patient as in Figs. 3c and 5b (left medial). **f** Same patient as in Figs. 4 and 5c (right perineal)

The BPD distribution was calculated from the PA images, and a corresponding heat map was created (Fig. 5a–c). The distribution of BPD showed inter-individual variation, but the maximum density within a 10-mm diameter region was approximately 30–100% in all cases (Table 2).

**Table 2:** Mean BPD values by area calculated

Patient No	Lower limb	Marked area (mean)	All areas (mean)	All areas (max)	All area—marked area (mean)	BPD ratio
1	Right medial	0.43	0.31	0.93	0.30	1.38
2	Left medial	0.41	0.28	0.87	0.26	1.48
3	Left medial	0.47	0.25	0.96	0.24	1.88
4	Right calf	0.44	0.21	0.88	0.20	2.05
	Left medial	0.37	0.32	0.92	0.31	1.17
5	Right medial	0.38	0.30	0.89	0.28	1.27
6	Left calf	0.18	0.15	0.49	0.14	1.22
7	Right medial	0.22	0.16	0.61	0.16	1.37
	Right calf	0.28	0.17	0.70	0.15	1.68
8	Left medial	0.18	0.17	0.77	0.17	1.07
	Right calf	0.40	0.29	0.82	0.29	1.36
9	Right medial	0.11	0.08	0.29	0.08	1.35

### Correlation analysis between high BPD regions and varicose vein detection regions in PA images

**Fig. 6:**
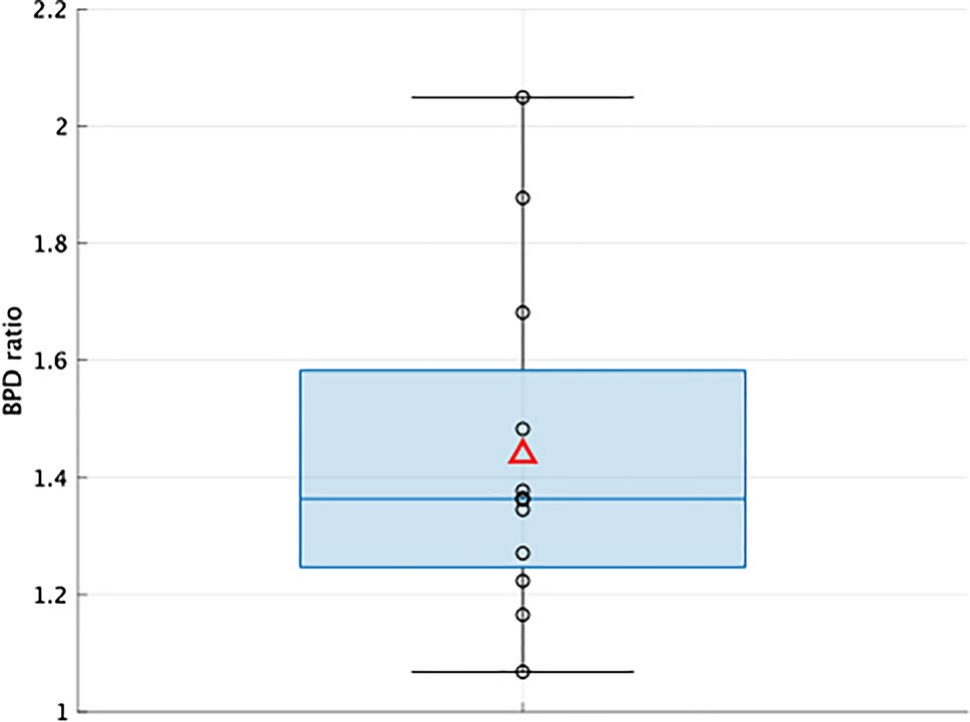
Box-and-whisker plot of BPD ratios for all cases. The top of the box is the third quartile (Q3), the bottom is the first quartile (Q1), the center is the median (Q2), the outside of the box is the whiskers, and the inside of the box is the mean. As can be seen from the diagram, the ratios are greater than 1 for all patients, and the mean, median, and quartile range are all greater than 1

The histogram of BPD was plotted using the relative probability. A higher density distribution was observed in the varicose vein detection regions than in other regions (Fig. 5d–f). Furthermore, the mean values of the BPD were compared, and in all 12 cases, the BPD in the varicose vein detection regions exceeded that in all areas (Table 2). The BPD ratio was defined as the mean BPD of the varicose vein detection area divided by that of the entire region. The ratio was found to be significantly higher than 1 (Fig. 6) (p value for one-sample one-tailed t-test, p = 0.000143).

## Discussion

This study demonstrated the capability of the PAI system to visualize, in three dimensions, vessels ranging from fine vessels invisible to the naked eye to dilated veins. Conventional clinical practice has relied on visual inspection for varicose veins and individual vessel assessment using ultrasonography. In contrast, the PAI system offers noninvasive visualization of the entire lower limb vasculature at a glance.

Furthermore, this study introduced BPD as a novel index to represent the proportion of vascular regions within a specified area. Analysis of lower limb images acquired via the PAI system revealed that the BPD in areas identified as varicose veins was higher compared to other regions. Visualizing BPD through a heat map enabled intuitive and clear depiction of the areas with elevated BPD levels. Therefore, the PAI system presents the potential for a new diagnostic imaging method for varicosities with quantitative properties. We hypothesized that the formation of varicose veins due to venous reflux disorders concurrently suggests an increase in the volume of blood within the pathway from the subcutaneous venous network to the deep veins. BPD has been introduced as an indicator to reflect this augmentation in blood volume. The higher BPD in areas visually identified as varicose veins compared to other regions may serve as evidence to support this hypothesis. In conclusion, the use of the PAI system could enhance the objectivity in assessing the “C” (clinical manifestation) and “A” (anatomic distribution) categories of CEAP classification. Moreover, it allows for the detection of finer vascular changes than those visible to the naked eye, which could be instrumental in identifying the early stages of varicose vein development, monitoring disease progression, and evaluating the effectiveness of treatments.

The PAI System is expected to be useful not only for more objective evaluation of “C” (clinical manifestation) and “A” (anatomic distribution) categories of the CEPA classification but also for determining the early stages of varicose vein development, disease progression, and treatment efficacy.

## Limitations

This study had several limitations. In this study, the camera and PA images were aligned; however, because the postures at the time of shooting were different, errors in the alignment remained. Thus, it is challenging to assert that the varicose veins identified by the physicians are perfectly congruent with the varicosities depicted in the PA images. These errors may result in lower BPD values even within regions identified by physicians as varicose veins. Therefore, enhancing the precision of the alignment is imperative, which is anticipated to improve the diagnostic capability of BPD.

Additionally, variations in posture during image acquisition may potentially affect the thickness of blood vessels. Since patients were in the supine or prone position when the PAI system was used in this study, the blood vessels may have been thinner than in the sitting or standing position during the actual diagnosis. It is necessary to investigate whether such differences in posture affect how varicose veins appear on PA images.

Optimization of the diameter of the circular area also may hold potential for augmenting the diagnostic accuracy of BPD. A circular area that is too small may merely blur the vascular image, thus only depicting local vascular density. Conversely, an excessively large circular area could impair the ability to discern local variations. The diameter of the circular area was set at 10 mm to facilitate the detection of vascular conditions, including not only dilated vessels but also reticular veins (vessel diameter 2–3 mm) and spider veins (vessel diameter less than 1 mm), as variations in the proportion of the vascular region within the circular area. This size was found to be effective as it allowed for the detection of spider-web patterns, which were also observable upon clinical examination, with a high BPD. Future studies should aim to increase the sample size and continue the search for an optimal circular area size to determine the most effective value.

In addition, varicose veins were assumed to be areas with high BPD; however, if it becomes necessary to distinguish these veins from clusters of web vessels or fine tortuous vessels, they may be identified by adding a curvature judgment.

Within the scope of this research, the difference between areas identified as varicose veins and other regions was calculated as the BPD ratio. The reason for using BPD ratio is the significant variability and individual differences in the BPD of areas not identified as varicose veins, which was observed to be a minimum value of 0.08 and a maximum value of 0.31 across the nine subjects and 12 limbs analyzed (Table 2).

In this study, the varicose vein area was determined based on the area specified by the physician, but further detection methods are needed to extract varicose vein areas from images in the future. Consideration of deviations from the mean or the use of AI for automatic segmentation into two groups may be necessary. In developing this, it is posited that including images of the healthy side in the analysis will be crucial for examining the balance between sensitivity and specificity in the extraction of varicose vein regions.

Moreover, this study was limited to a small cohort of only nine patients, which may have been insufficient for analyzing the correlation between the CEAP classification and the BPD ratio. Further investigation is essential to stratify the CEAP severity within individual lower limbs and to explore the association with the “P” (pathophysiologic) category of the CEAP classification, which is only verifiable through ultrasound. This necessitates the inclusion of a larger patient population, encompassing those with healthy control groups as well.

### Future work

**Fig. 7:**
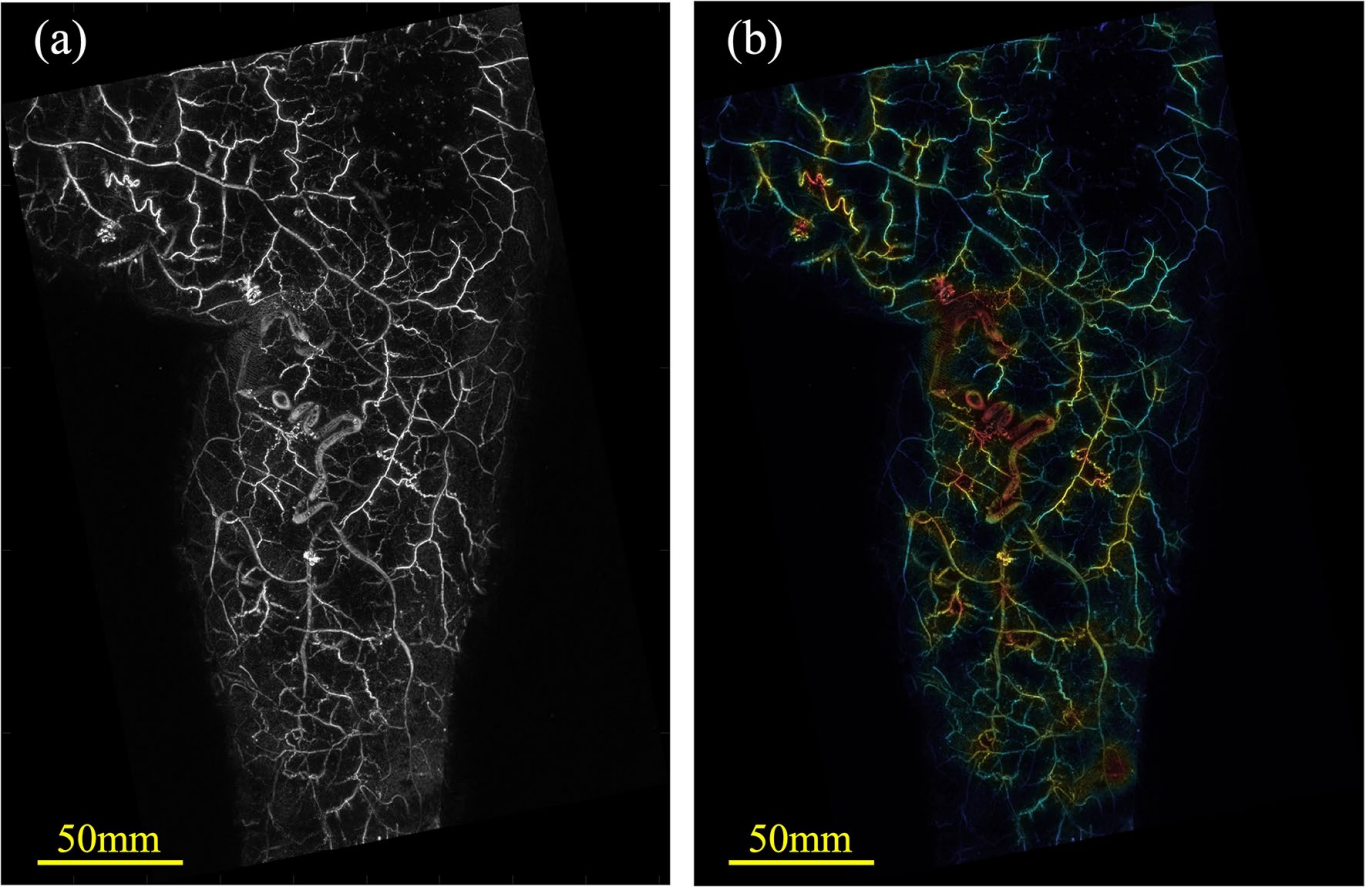
Example of a PA image and varicose vein display. **b** Example of another colored BPD map for varicose vein generated from PA image (**a**)

The lower limb images acquired via PAI allowed visualization of many vessels that could not be confirmed by visual inspection, and the course of the vessels could be observed in more detail (Fig. 7a). The BPD heat map created in this study revealed the existence of high BPD regions not only in the marked varicose vein sites but also in other locations. By detecting changes in vessels that are not visible to the eye, such as thin or deep-running vessels, this study may contribute to the early detection of lower limb varicose veins. Various display methods using BPD (Figs. 5b, 7b) indicated the potential to contribute to diagnostic support, such as reducing the interobserver variability of visual inspection and enabling the early detection of varicose veins. Moreover, quantification of BPD is expected to be useful in various situations such as evaluating treatment effects and performing screening tests.

## Conclusions

In this study, we showed that lower limb images acquired using the PA imaging system revealed the existence of vessels ranging from thin ones that were not visible to the eye to thick ones that were expanded and varicose veins in three dimensions. We demonstrated that the regions where varicose veins were identified had a significantly higher BPD in the lower limb images acquired using the PAI system. These results suggest that PA imaging could provide useful information for the diagnosis and treatment of lower limb varicose veins and contribute to the improvement of quality of life.

## Data Availability

The data are not publicly available due to restrictions; they contain information that could compromise the privacy of the research participants.
